# Rootstock effects on scion gene expression in maritime pine

**DOI:** 10.1038/s41598-021-90672-y

**Published:** 2021-06-02

**Authors:** M. López-Hinojosa, N. de María, M. A. Guevara, M. D. Vélez, J. A. Cabezas, L. M. Díaz, J. A. Mancha, A. Pizarro, L. F. Manjarrez, C. Collada, C. Díaz-Sala, M. T. Cervera Goy

**Affiliations:** 1 Departamento de Ecología y Genética Forestal, Centro de Investigación Forestal (CIFOR), Instituto Nacional de Investigación y Tecnología Agraria y Alimentaria (INIA), Madrid, Spain; 2 Unidad Mixta de Genómica y Ecofisiología Forestal, Instituto Nacional de Investigación y Tecnología Agraria y Alimentaria (INIA)/Universidad Politécnica de Madrid (INIA/UPM), Madrid, Spain; 3 Departamento de Ciencias de la Vida, Universidad de Alcalá (UAH), Alcalá de Henares, Spain; 4 Departamento de Sistemas y Recursos Naturales, E.T.S.I. Montes, Forestal y Medio Natural, Universidad Politécnica de Madrid (UPM), Madrid, Spain

**Keywords:** Genetics, Molecular biology, Plant sciences

## Abstract

Pines are the dominant conifers in Mediterranean forests. As long-lived sessile organisms that seasonally have to cope with drought periods, they have developed a variety of adaptive responses. However, during last decades, highly intense and long-lasting drought events could have contributed to decay and mortality of the most susceptible trees. Among conifer species, *Pinus pinaster* Ait. shows remarkable ability to adapt to different environments. Previous molecular analysis of a full-sib family designed to study drought response led us to find active transcriptional activity of stress-responding genes even without water deprivation in tolerant genotypes. To improve our knowledge about communication between above- and below-ground organs of maritime pine, we have analyzed four graft-type constructions using two siblings as rootstocks and their progenitors, Gal 1056 and Oria 6, as scions. Transcriptomic profiles of needles from both scions were modified by the rootstock they were grafted on. However, the most significant differential gene expression was observed in drought-sensitive Gal 1056, while in drought-tolerant Oria 6, differential gene expression was very much lower. Furthermore, both scions grafted onto drought-tolerant rootstocks showed activation of genes involved in tolerance to abiotic stress, and is most remarkable in Oria 6 grafts where higher accumulation of transcripts involved in phytohormone action, transcriptional regulation, photosynthesis and signaling has been found. Additionally, processes, such as those related to secondary metabolism, were mainly associated with the scion genotype. This study provides pioneering information about rootstock effects on scion gene expression in conifers.

## Introduction

According to the latest report of the Intergovernmental Panel on Climate Change^1^ the Mediterranean area is one of the most vulnerable regions to the impacts of global warming, suffering recurrent drought periods caused by the increase in temperature and the decrease in rainfall rates, coupled to torrential rainfall events. For all the above reasons, adaptation of Mediterranean forests to face these environmental changes are most important to secure their survival and performance. Mediterranean conifers are long-lived organisms which have developed specific mechanisms to respond and survive to recurrent drought events, which have been described at molecular, cellular, and physiological levels^2^. Conifers are considered more drought resistant than angiosperms mainly due to their xylem, which is made of tracheids, and needles which have a singular hydraulic function^3^. Additionally, other anatomical structures play an important role in drought response by affecting water potential, net photosynthetic, transpiration and cavitation rates, stomata conductance and carboxylation efficiency^4–6^. Drought-induced structural changes can have long-lasting effects on root mass^7^, xylem^8,9^, cell size^10^, lumen and cell wall anatomical characteristics^7^. From a molecular perspective, drought perception and its initial signaling in trees involves calcium-dependent signaling and mitogen-activating protein kinases (MAPKs)^11^. Drought tolerance results from a combination of processes: decrease of leaf/needle water potential induces stomatal closure due to the accumulation of the phytohormone abscisic acid (ABA)^12^, thus controlling transpiration and preventing hydraulic failure. Additionally, accumulation of osmolytes, that stabilizes macromolecules and cellular components such as membranes, as well as activation of antioxidant systems avoid cellular damage^13,14^. To avoid carbon starvation caused by the decrease in photosynthesis due to stomatal closure, an efficient management of carbon reserves is associated with the increase of non-structural carbohydrate content in response to drought duration^15^. In drought tolerance processes, phytohormones such as abscisic acid (ABA), salicylic acid (SA), jasmonic acid (JA), ethylene (ET), auxins, gibberellins (GA), cytokinins (CK) and brassinosteroids (BRs) are growth regulators involved in drought stress signaling, cross talking among themselves and/or with other factors^16–18^.

Maritime pine (*Pinus pinaster* Ait.) is a dominant autochthonous tree species in western Mediterranean forests. Found in different ecosystems, maritime pine is a key component of arid areas. In addition to its ecological importance, it has a high socio-economic value*,* being one of the main sources of forest biomass, due to its use in plantations and reforestation of large areas. It is also one of the major resins producing species. It is characterized by a high genetic and adaptive variability^19^. Although Maritime pine is considered a drought-avoiding species due to its high stomatal sensitivity to soil water deficit^20^, the species shows intraspecific variability in multiple functional traits related to drought adaptation and tolerance^21–26^. Preliminary studies found genes expressed in a time- and organ-dependent manner analyzing aerial organs and roots in response to drought^27^. *Pinus pinaster* shows increased recalcitrance to vegetative propagation by cuttings during the first five years. For this reason, maritime pine seed orchards are based on intraspecific grafts of selected trees as scions onto uncontrolled rootstocks^28^. This asexual propagation method, based in the combination of two genotypes, is an ancient agronomic technique that has been routinely used to enhance tolerance to stress, improve quality and increase production of woody and non-woody species^29,30^. Thus, grafting has been used to improve drought tolerance of a wide variety of fruit trees^31–33^ and herbaceous species^34,35^. In forest tree species, the use of drought tolerant rootstocks has improved drought response of the whole plant^36^. Grafts are designed to combine traits of selected scions and rootstocks. Scions determine shoot growth while rootstocks control root growth determining the uptake of below-ground resources^37^. Thus, drought-tolerant rootstocks enhance root development, improving water availability^37,38^, and increase hydraulic conductivity reducing cavitation of grafted scions^39,40^, that, together with root-to-shoot-transported signal molecules, may modify photosynthesis and stomatal conductance^41^. Recently, grafting has been used to study the long-distance communication between organs^42^. However, most of the grafting studies have been focused on flowering plants, and due to the divergence of angiosperms and gymnosperms (350 Myr ago), and the existing anatomical differences, functional information cannot be transferred to conifers.

The rapid evolution of transcriptomics and functional genomics tools, such as high-throughput sequencing technologies, has enabled the study of the biological mechanisms underlying adaptive and productive traits in conifers. Several RNA-Seq analysis have been performed and thematic databases have been developed such as TreeGenes (https://treegenesdb.org/), Gymno PLAZA (https://bioinformatics.psb.ugent.be/plaza/versions/gymno-plaza/), CONGENIE (http://congenie.org/) and SustainPineDB (http://www.scbi.uma.es/sustainpinedb/). Transcriptomic analysis of *P. pinaster* drought response of drought-tolerant and drought-sensitive trees revealed that tolerant individuals are pre-adapted for coping with drought by constitutively expressing stress-related genes, which are only detected in sensitive individuals during the late stage of the drought response^43^. In this study we designed intraspecific grafts of *P. pinaster* using as scions and rootstocks both drought-tolerant and drought-sensitive maritime pine genotypes, to analyze the effect of rootstocks on the needle transcriptome of the scions.

## Results

### RNA sequencing and transcriptomic profiles

**Figure 1:**
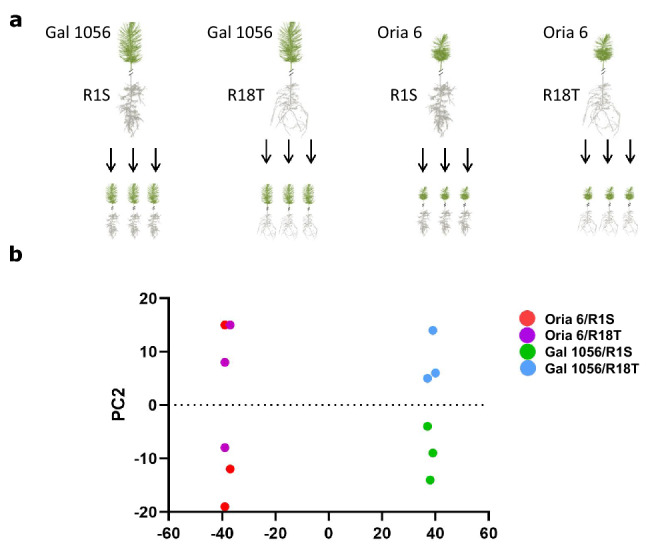
Grafts analyzed combining drought-tolerant (T: R18T) and drought-sensitive (S: R1S) rootstocks with drought-tolerant (O: Oria 6) and drought-sensitive (G: Gal 1056) scions. (**a**) Experimental design, (**b**) PCA plot of Log2-normalized FPKM of the 12 analyzed RNASeq samples.

Three needle biological replicates of each of the four constructs were processed to build 12 cDNA libraries (Fig. 1a). A total of 206.978 M paired 75 bp-long reads were obtained, ranging from 12.6 to 28.3 M raw reads per library. Pre-processing led to 4.9 to 24.1 M (38.7 to 85%) high-quality reads per library. Filtered reads were independently mapped against the *Pinus pinaster* reference transcriptome (ProCoGen, http://www.procogen.eu), with an average mapping rate of 91% and 12,042,168 mapped reads (Supplementary Table 1). A total of 75,507 sequences were annotated by BLASTX to the UniProtKB/Swiss-Prot and RefSeq databases, of which 64,263 were annotated with 681,099 gene ontology (GO) terms and 41,560 shown significant similarity to known domains.

Initially, principal component analysis (PCA) of expression data showed clustering of analyzed samples, where the first principal component (PC1), associated with scion genotype, explained 84% of the variance, while rootstock genotypes onto which each scion was grafted (PC2) explained 8% of the variance (Fig. 1b).

### Analysis of DEGs

In order to explore if genetically related rootstocks, showing contrasting responses to drought, may modify transcript profiles of scion needles under well-watered conditions, genes were tested for differential expression. Two differential gene expression analyses were carried. The first comparison was performed to analyze if drought-tolerant or drought-sensitive rootstocks may modify the transcriptomic profile of grafted drought-sensitive (Gal 1056/R1S vs. Gal 1056/R18T) and drought-tolerant (Oria 6/R1S vs. Oria 6/R18T) scions. A second comparison was carried out between transcriptomes of drought-tolerant and drought-sensitive scions to analyze how they differ when drafted onto drought-tolerant (Gal 1056/R18T vs. Oria 6/R18T) and drought-sensitive (Gal 1056/R1S vs. Oria 6/R1S) rootstocks. Additionally, differentially expressed genes were further analyzed by GO, MapMan terms and KEGG categorization.

### Genes differentially expressed in the needles of *P. pinaster* grafts associated to a rootstock effect

#### Analysis of the rootstock effect on drought-sensitive scions (Gal 1056/R1S vs. Gal 1056/R18T)

**Figure 2:**
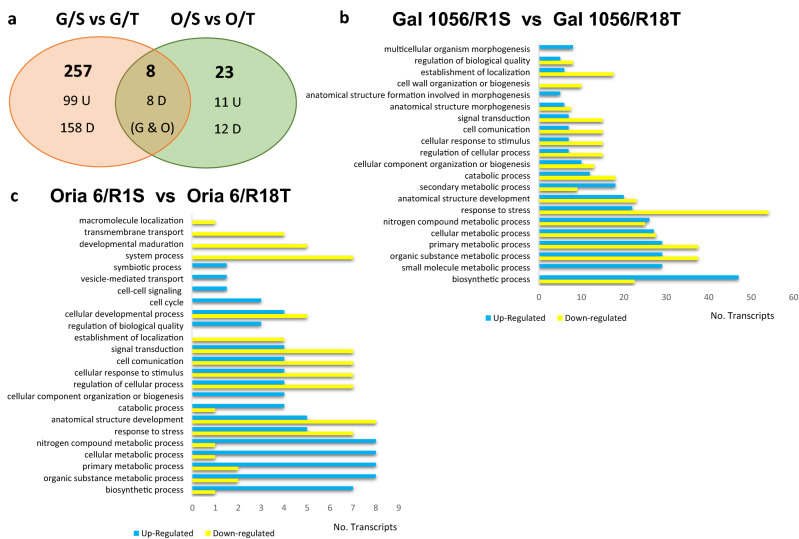
DEGs identified in needles of drought-sensitive (G: Gal 1056) or drought-tolerant (O: Oria 6) scions grafted onto drought-sensitive (S: R1S) versus drought-tolerant (T: R18T) rootstocks. (**a**) Venn diagram showing DEGs [Log2FC ≤ − 1.5 and Log2FC ≥ 1.5] for upregulated (U) and downregulated DEGs (D), respectively. (**b**–**c**) Histogram presentation of GO terms enriched in significantly upregulated (blue) and downregulated (yellow) genes exclusively differentially expressed in G/S versus G/T (**b**) or O/S versus O/T (**c**) scions. *P* values (*P* < 0.05) were calculated according to the Wald test.

The transcriptome analyses revealed 257 differentially expressed genes (DEGs) exclusively detected in needles of Gal 1056/R1S versus Gal 1056/R18T, where 99 and 158 were upregulated and downregulated, respectively (Fig. 2a). The list of the genes is provided in Supplementary Table 2.

Since the most informative GO category in terms of assignments was biological process, the analysis of their GO terms distribution revealed that DEGs associated with “Biosynthetic process” (14%), was the most significantly upregulated in Gal 1056/R18T, while those DEGs classified in “Response to stress” (14%), were significantly down regulated (Fig. 2b). MapMan analysis showed that categories “Flavonoid biosynthesis” and “Fatty acid biosynthesis” were enriched in upregulated DEGs, while “External stimuli response”, “Protein homeostasis” were highly enriched in downregulated ones (Supplementary Table 2).

**Figure 3:**
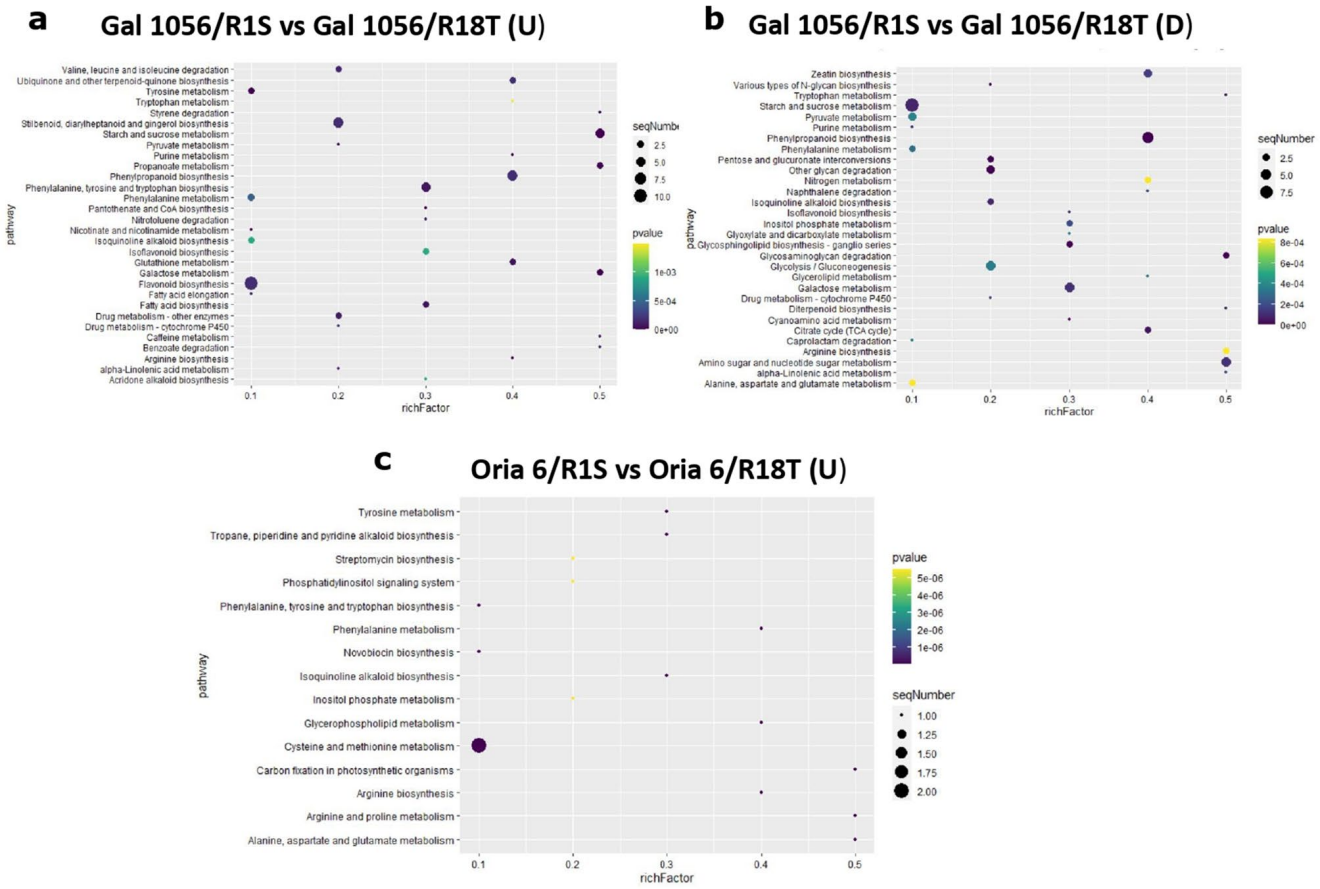
Top 30 enriched KEGG pathways analysis of (**a**) significantly upregulated (≥ 1.5 Log2FC) and (**b**) downregulated (≤ − 1.5 Log2FC) genes exclusively differentially expressed in Gal 1056/R1S versus Gal 1056/R18T. (**c**) Upregulated (≥ 1.5 Log2FC) genes exclusively differentially expressed in Oria 6/R1S versus Oria 6/R18T. Rich factor represents the ratio of the DEG number and the number of all genes in the pathway. *P* values (*P* < 0.05) were calculated according to the Wald test.

KEGG analysis of the top 30 enriched pathways revealed that “Flavonoid biosynthesis”, “Phenylpropanoid biosynthesis”, “Stilbenoid, diarylheptanoid and gingerol biosynthesis”, and “Starch and sucrose metabolism” were the processes with the highest number of significantly upregulated DEGs in Gal 1056/R18T (Fig. 3a). This analysis also reported that “Starch and sucrose metabolism”, “Phenylpropanoid biosynthesis” were the categories with the highest number of significantly downregulated DEGs (Fig. 3b).

**Figure 4:**
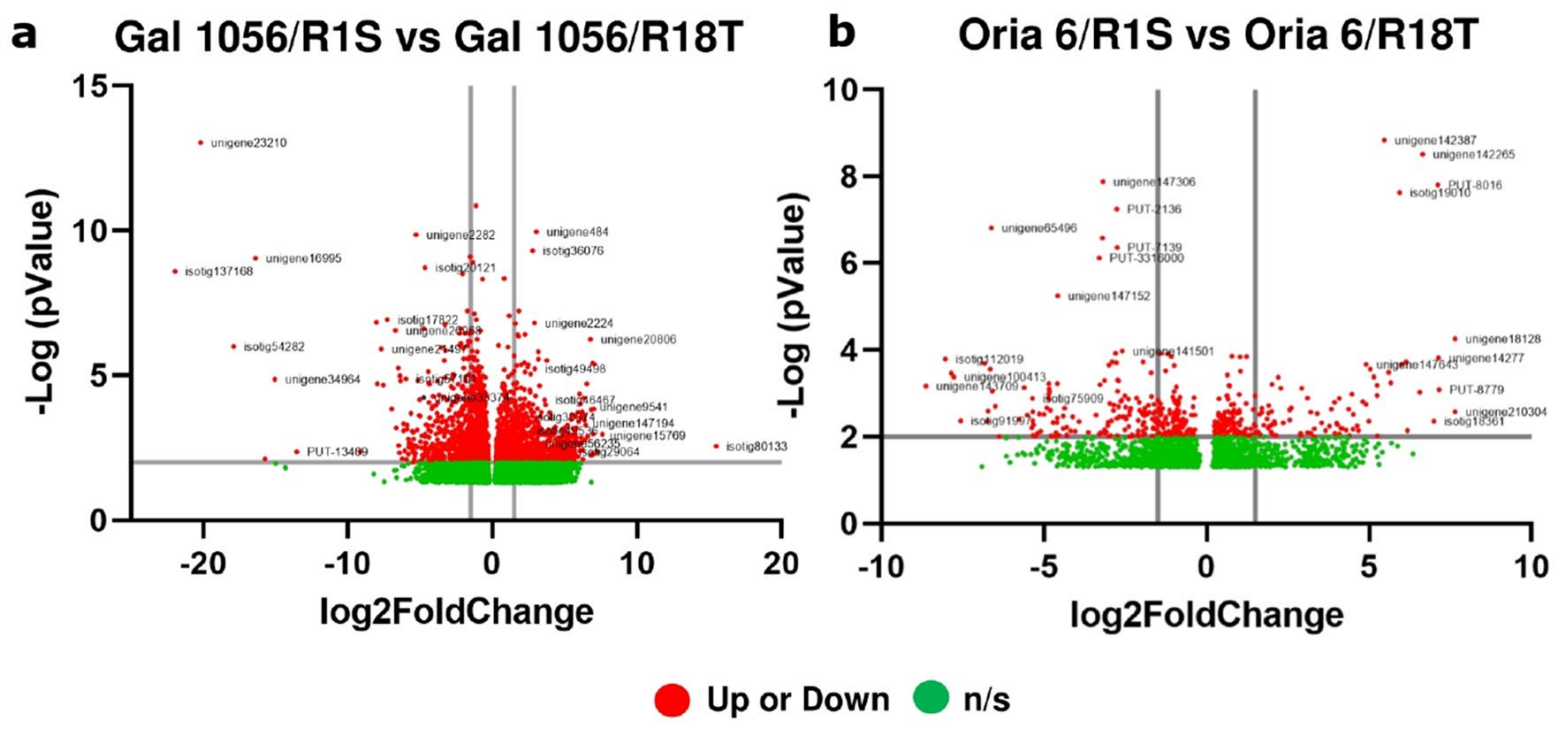
Volcano plots of differential expression profiles comparing genes differentially expressed in needle scions of (**a**) Gal 1056/R1S versus Gal 1056/R18T and (**b**) Oria 6/R1S versus Oria 6/R18T grafts (up or down: significantly upregulated or downregulated genes [Log2FC ≤ − 1.5 and Log2FC ≥ 1.5]; n/s: no significant genes). The list of the genes is provided in Supplementary Table 3 and Supplementary Table 4. *P* values (*P* < 0.05) were calculated according to the Wald test.

Among the most upregulated DEGs in Gal 1056/R18T, MYB transcription factor (TF/TFs) (unigene15769) showed the highest Log2FC value (7.6). In addition, genes encoding anthocyanidin synthases, anthocyanidin reductases, chalcone synthases and flavonone 3′ 5′-hydroxylases (isotig30374, isotig49536, unigene56235, isotig49498, isotig29064, unigene2224, unigene147194); aerogenate dehydratase (isotig46467); and protein phosphatase 2C (PP2C; unigene9541), were also found to be significantly upregulated (Fig. 4a, Supplementary Table 2 and 3). Contrastingly, 26S proteasome regulatory subunit RPN2a (unigene34964); AGP beta-1,3-galactosyltransferase (isotig17822), and dehydration-responsive protein RD22 and defensin-1 (unigene26968, isotig57101, unigene35374) were the most downregulated genes (Fig. 4a, Supplementary Table 3).

#### Analysis of the rootstock effect on drought-tolerant scion (Oria 6/R1S vs. Oria 6/R18T)

The gene expression analysis of Oria6 needles grafted onto drought-sensitive (R1S) versus drought-tolerant (R18T) rootstocks resulted on 31 DEGs, 23 of them exclusively detected in Oria6, where 11 were upregulated and the remaining 12 were downregulated (Fig. 2a). The list of the genes is provided in Supplementary Table 2.

The analysis of GO terms distribution showed that “Nitrogen compound metabolic process” (9%), “Organic substance metabolic process” (9%), “Primary metabolic process” (9%), and “Cellular metabolic process” (9%) were the most significant biological processes in upregulated DEGs, while “Anatomical structure development” (10%) and “Response to stress” (9%), were the most significantly downregulated (Fig. 2c).

MapMan analysis revealed that categories “Photosynthesis” and “Protein biosynthesis were enriched in upregulated DEGs, whereas “RNA biosynthesis” and “Cell cycle organization” were enriched in upregulated and downregulated DEGs (Supplementary Table 2).

Top KEGG pathways analysis showed that “Cysteine and methionine metabolism” was the category with the highest number of significantly upregulated DEGs (Fig. 3c) whereas KEGG results did not reveal significantly downregulated ones.

Among significantly upregulated DEGs in Oria 6/R1S versus Oria 6/R18T, myo- inositol-1-phosphate phosphatase (unigene18128), showed the highest Log2FC (7.6). Two significantly upregulated TFs were a C2H2-ZF (PUT-8016) and a bZIP (unigene142265). Additionally, ribosomal protein L25/L23 (isotig19010) and prephenate aminotransferase (PPA-AT; unigene142387) were also upregulated (Fig. 4b, Supplementary Table 4). Significantly downregulated DEGs included peroxisomal membrane protein (unigene65496) as well as genes encoding GRAS (isotig113077) and several CAMTA (unigene147152, PUT-3316000, unigene147128, unigene147306, PUT-2136, PUT-7139) TFs.

#### Needle DEGs shared between scions grafted onto drought-sensitive versus drought-tolerant rootstocks

Only 8 downregulated genes were significantly differentially expressed in both scions grafted onto drought-sensitive (R1S) versus drought-tolerant (R18T) rootstocks (Fig. 2a). MapMan analysis showed that “RNA biosynthesis” was the significantly enriched category in DEGs, with downregulated genes encoding CAMTA transcription factors (unigene33819, PUT-9211, unigene20892).

### Genes differentially expressed in needles of *P. pinaster* scions grafted onto drought- sensitive or drought- tolerant rootstocks

#### Analysis of the gene expression between drought-sensitive and drought-tolerant scions grafted onto drought-sensitive rootstocks (Gal 1056/R1S vs. Oria 6/R1S).

**Figure 5:**
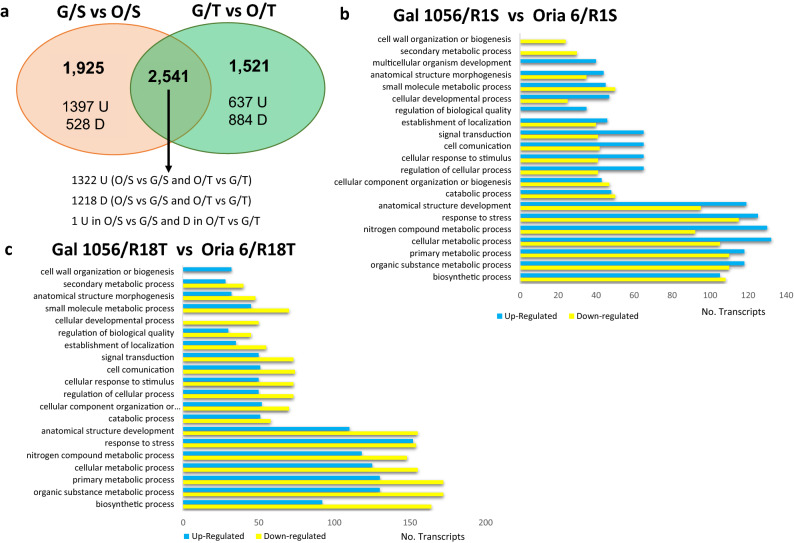
DEGs identified in needles of drought-sensitive (G: Gal1056) versus drought-tolerant (O: Oria6) scions grafted onto drought-sensitive (S: R1S) or drought-tolerant (T: R18T) rootstocks. (**a**) Venn diagram showing DEGs [Log2FC ≤ − 1.5 and Log2FC ≥ 1.5] for upregulated (U) and downregulated genes (D), respectively. (**b**–**c**) Histogram presentation of GO terms enriched in significantly upregulated (blue) and downregulated (yellow) genes exclusively differentially expressed in G/S versus O/S (**b**) and G/T versus O/T (**c**) scions. *P* values (*P* < 0.05) were calculated according to the Wald test.

A total of 1925 DEGs were exclusively identified in needles of Gal 1056/R1S versus Oria 6/R1S, where 1397 are upregulated and 528 downregulated (Fig. 5a).

The analysis of the top 20 most significant GO terms showed that “Cellular metabolic process” (9%), “Nitrogen compound metabolic process” (9%), and “Response to stress” (8%) were the biological processes with higher number of upregulated DEGs in Oria 6/R1S (Fig. 5b). The processes enriched with high number of significantly downregulated DEGs were “Response to stress” (9%), “Organic substance metabolic process” (9%), and “Primary metabolic process” (9%) (Fig. 5b).

MapMan analysis showed that “External stimuli response” and “Protein modification” were categories with significantly enriched in upregulated genes in Oria 6/R1S grafts (Log2FC ≥ 1.5); whereas, “Secondary metabolism” was significantly enriched in upregulated genes in Gal 1056/R1S (Log2FC ≤ − 1.5) (Supplementary Table 5). “RNA biosynthesis” and “Phytohormone action” processes included high number of upregulated and downregulated DEGs.

**Figure 6:**
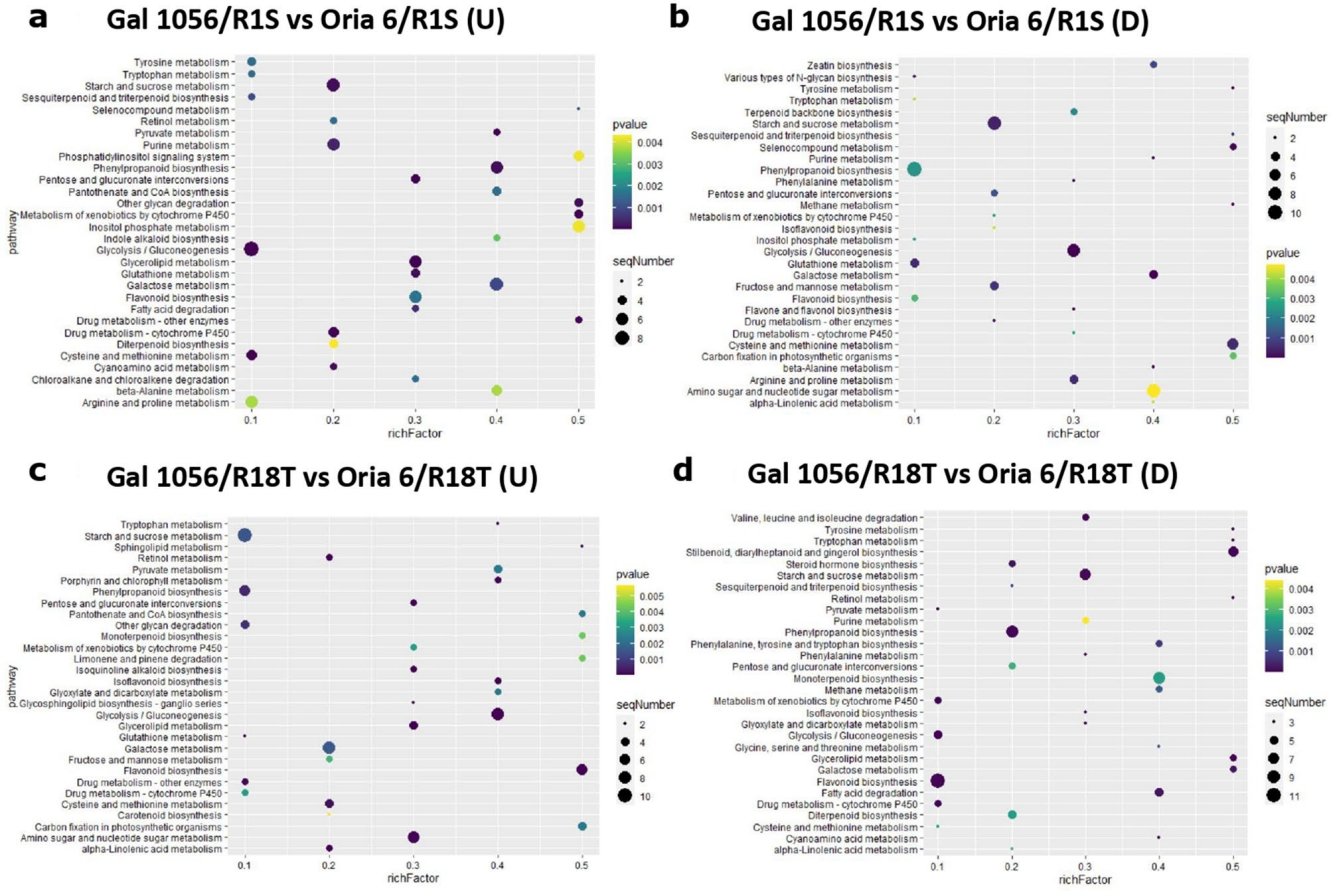
Top 30 enriched KEGG pathways analysis. (**a**) Significantly upregulated (≥ 1.5 Log2FC) and (**b**) downregulated (≤ − 1.5 Log2FC) genes exclusively differentially expressed in Gal 1056/R1S versus Oria 6/R1S. (**c**) Significantly upregulated (≥ 1.5 Log2FC) and (**d**) downregulated (≤ − 1.5 Log2 FC) genes exclusively differentially expressed in Gal 1056/R18T versus Oria 6/R18T. Rich factor represents the ratio of the DEGs number and the number of all genes in the pathway. *P* values (*P* < 0.05) were calculated according to the Wald test.

Among the top 30 most significant KEGG pathways “Phenylpropanoid biosynthesis”, “Glycolysis/Gluconeogenesis” and “Starch and sucrose metabolism” were the categories with the highest number of significantly upregulated DEGs in Oria 6/R1S (Fig. 6a). This analysis also showed that the latter two categories also included the highest number of significantly upregulated genes in Gal 1056/R1S (Fig. 6b).

**Figure 7:**
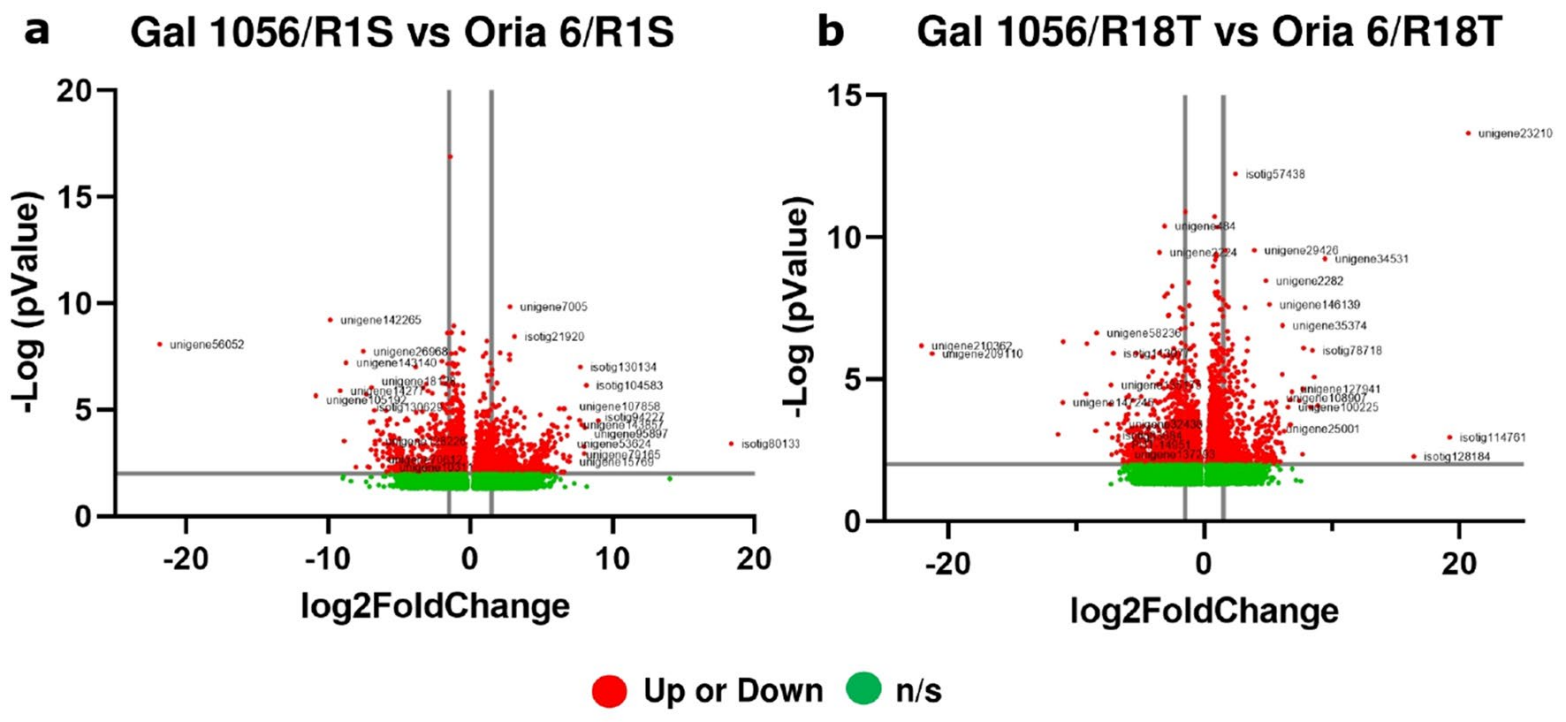
Volcano plots of differential expression profiles comparing genes differentially expressed in needle scions of (**a**) Gal 1056/R1S versus Oria 6/R1S and (**b**) Gal 1056/R18T versus Oria 6/R18Tgrafts (up or down: significantly upregulated or downregulated genes [Log2FC ≤ − 1.5 and Log2FC ≥ 1.5]; n/s: no significant genes). Details of the genes have been given in Supplementary Table 6 and Supplementary Table 7. *P* values (*P* < 0.05) were calculated according to the Wald test.

In particular, significant upregulated expression levels of protein phosphatase 2C (isotig80133); component CPFS6/CFIm68 (isotig94227); leucoanthocyanidin dioxygenase (unigene143857) and ubiquitin carboxyl-terminal hydrolase 54 (unigene95897) were observed in Oria 6/R1S. Other DEGs, such as histone deacetylase 15 (unigene107858), as well as MYB (unigene15769) and ERF (unigene53624) TFs were also upregulated (Fig. 7a, Supplementary Table 6). Among downregulated DEGs, chlorophyll a-b binding protein type II 2 (unigene56052), component of the photosystem II involved in photophosphorylation showed the lowest Log2FC value (− 21.8). Additionally, two component RPL21(unigene105192, isotig130629) and rRNA processing factor (IRP1) (unigene143140); 2-isopropylmalate synthase (unigene14277); dehydration-responsive protein RD22 (unigene26968); and bZIP (unigene142265), C2H2 (PUT-8016) and NAC (unigene10311) TFs, showed higher levels of transcript accumulation in Gal 1056/R1S (Fig. 7a, Supplementary Table 6).

#### Analysis of gene expression between drought-sensitive and drought-tolerant scions grafted onto drought-tolerant rootstocks (Gal 1056/R18T vs. Oria 6/R18T)

Transcriptome analysis showed 1521 DEGs exclusively identified in needles from Gal 1056/R18T grafts compared to Oria 6/R18T plants, where 637 are upregulated and 884 downregulated (Fig. 5a).

Among the top-20 most significant GO terms we found “Response to stress” (11%) to be the biological process more significantly enriched in upregulated DEGs in Oria 6/R18T (Fig. 5c); while “Primary metabolic process” (9%) and “Organic substance metabolic process” (9%) were the most significantly upregulated in Gal 1056/R18T (Fig. 5c).

MapMan categorization showed that “Protein homeostasis” was significantly enriched in upregulated DEGs (Log2FC ≥ 1.5) in Oria 6/R18T, whereas categories “Secondary metabolism” and “Lipid metabolism were significantly enriched in downregulated DEGs (Log2FC ≤ − 1.5) (Supplementary Table 5). “RNA biosynthesis”, “Phytohormone action”, “Photosynthesis” and “Cellular respiration” were highly enriched in upregulated and downregulated DEGs. Within DEGs classified in “Secondary metabolism”, genes involved in betaine and glucosinolate biosynthesis were upregulated, whereas most of genes involved in phenolics and terpenoids biosynthesis were downregulated and therefore upregulated in Gal 1056/R18T.

The top 30 KEGG pathway analysis showed that “Glycolysis/Gluconeogenesis”, “Amino sugar and nucleotide sugar metabolism” and “Flavonoid biosynthesis” were the categories significantly enriched in upregulated genes in Oria 6/R18T (Fig. 6c). “Flavonoid biosynthesis”, “Phenylpropanoid biosynthesis”, and “Starch and sucrose metabolism” were the categories significantly enriched in upregulated genes in Gal 1056/R18T (Fig. 6d).

Elongation factor 1-alpha (isotig114761), involved in RNA polymerase II-dependent transcription elongation, showed the highest Log2FC value (19.2) among the most significantly upregulated genes in Oria 6/R18T. Also, two ubiquitin-fold proteins (UBQ, isotig128184, isotig78718); RNA editing factor (ORRM-type) (unigene127941); two receptor proteins (GID1) (unigene108907, unigene25001); and D-lactate dehydrogenase (unigene100225) were also significantly upregulated in Oria 6/R18T (Fig. 7b, Supplementary Table 7). By contrast, the most significantly downregulated DEG was chalcone synthase (CHS) (unigene147245), involved in the secondary metabolism of flavonoids with the lowest Log2FC value (− 11.04). Other genes, such as serine hydroxymethyltransferase (SHM) (isotig83684); component PetG/V (unigene58236); GRAS (isotig113077) and MYB (unigene32436) TFs; tubulin beta-1/beta-2 chain (unigene137293); and 27 kDa Golgi SNARE protein (PUT-14951) were also upregulated in Gal 1056/R18T (Fig. 7b, Supplementary Table 7).

#### Genes significantly expressed in needles from drought-sensitive and drought-tolerant scions independently of the rootstock genotype to which they were grafted

The comparative analysis between genes significantly expressed in needles from drought-sensitive and drought-tolerant scions grafted onto sensitive (Gal 1056/R1S vs. Oria 6/R1S) and tolerant (Gal 1056/R18T vs. Oria 6/R18T) rootstocks revealed 2541 DEGs shared, 1322 of them were upregulated, while 1218 were downregulated (Fig. 5a). The single DEG that showed upregulation in the comparison of scions grafted onto drought-sensitive rootstocks, while upregulation in the comparison of scions grafted onto the drought-tolerant rootstocks, could not be annotated.

The analysis of the top 20 most significant GO terms showed that “Response to stress” (9%), “Cellular metabolic process” (8%), were the biological processes significantly upregulated in Oria 6 (Supplementary Fig. S2), and “Cellular metabolic process” (9%) was the most significantly upregulated in Gal 1056 (Supplementary Fig. S2) regardless the rootstock. This analysis also showed that DEGs classified in “Macromolecule localization” (2%) and “Multicellular organism development” (1%) were only found upregulated in Oria 6 regardless the rootstock (Supplementary Fig. S2) while DEGs in “Secondary metabolic process” (2%) and “Cell cycle” (2%) were only upregulated in Gal 1056 regardless the rootstock.

Significantly upregulated DEGs included bZIP (unigene128170), DREB (unigene20075), and heat shock TFs (isotig85377, unigene31301, unigene146137, isotig63123, unigene52857, unigene129711, unigene33094, isotig58263); chlorophyll a–b binding protein type 2 member 1A and component PsbR (unigene207985, unigene146225); S-nitrosothiol reductases (TRX5) (PUT-13308, isotig85683); pre-mRNA-processing protein (LUC7) (isotig26861); and component SKP2 and Cullin-1 (isotig49845, unigene133439). Contrastingly, significantly downregulated DEGs included genes involved in the biogenesis of ribosomes (isotig79958, unigene129248); histone (H2A) (isotig10673); PHD zinc fingers (PUT-12952, unigene129455); C2H2 (unigene16552), ERF (unigene20499) and HSF (isotig104905) TFs; Pol I-V shared regulatory subunit 12 (unigene18172); and abscisic stress-ripening protein 1 (unigene146238).

### Gene expression analysis by quantitative real-time PCR

Expression analysis of five DEGs was performed on three biological replicates from each of the four grafts by qRT-PCR, in order to validate RNASeq analysis. DREB TF (DREB), pre-mRNA-processing protein (LUC7), outer mitochondrion membrane TOM translocation system (TOM), C2H2-ZF TF (C2H2-ZF), and macrophage migration inhibitory factor (MIF) were analyzed in needles of sensitive and tolerant plants. The relative quantification of all these DEGs showed results in agreement with the transcriptomic analysis (Supplementary Fig. S1).

## Discussion

Grafting has been broadly used to propagate fruit trees and vegetables and to study different aspects of plant biological research, such as systemic signaling. However, its use in forest trees research in general, and in conifer research in particular, has been scarce^44^. A limitation of its application in some conifer species is the loss of vegetative propagation capacity associated with age and maturation. The rate and extent of rooting capacity is species-dependent. Loss of rooting ability occurs early in *Pinus pinaster*, which limits the production of rootstocks. Due to the limitation in the number of rootstocks, in this study, progeny individuals from a controlled full-sib cross were used as rootstocks and their progenitors as scions to improve graft compatibility^45^.

**Figure 8:**
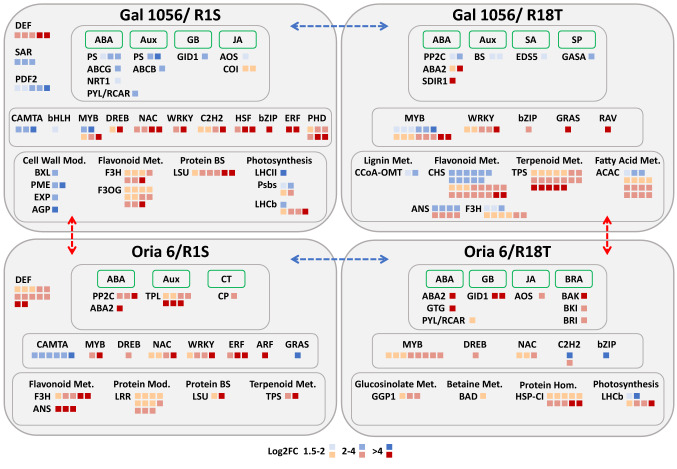
A schematic model of differential expression profiles comparing genes significantly differentially expressed in needle scions of Gal 1056/R1S, Gal 1056/R18T, Oria 6/R1S and Oria 6/R18T (≤ − 1.5 and ≥ 1.5 Log2FC) plants. Blue arrows indicate G/S versus G/T and O/S versus O/T comparisons that allowed identification of representative exclusively differentially expressed genes (blue filled squares). Red arrows indicate G/S versus O/S and G/T versus O/T comparisons that allowed identification of representative exclusively differentially expressed genes (red filled squares). Gray squares group phytohormones, TFs and biological processes. *ABA* abscisic acid, *ABA2* xanthoxin oxidase, *ABCB* ATP-binding cassette subfamily B transporter, *ABCG* ATP-binding cassette subfamily G transporter, *ACAC* acetyl-CoA carboxylase, *AGP* arabinogalactan protein, *ANS* anthocyanidin synthase, *AOS* allene oxidase synthase, *ARF* auxin response factor, *Aux* auxin, *BAD* betaine-aldehyde dehydrogenase, *BAK* brassinosteroid co-receptor protein kinase, *bHLH* basic helix-loop-helix, *BKI* brassinosteroid receptor kinase inhibitor, *BRA* brassinosteroid, *BRI* brassinosteroid receptor protein kinase, *BS* biosynthesis, *BXL* β-xylosidase, *bZIP* Basic Leucine Zipper Domain, *CAMTA* Calmodulin-binding transcription activator, *CCoA-OMT* caffeoyl-CoA 3-O-methyltransferase, *CHS* chalcone synthase, *C2H2* C2H2 zinc finger, *COI* jasmonic acid component COI, *CP* cytokinin phosphoribohydrolase, *CT* cytokinin, *DEF* defense mechanism, *DREB* dehydration-responsive element binding protein, *EDS5* salicylic acid transporter, *ERF* Apetala 2/ethylene responsive factor, *EXP* expansin, *F3H* flavanone 3-hydroxylase, *F3OG* flavonol-3-O-glycoside rhamnosyltransferase, *GASA* GASA-precursor polypeptide, *GB* gibberellin, *GGP1* gamma-glutamyl peptidase, *GID1* gibberellin receptor protein, *GRAS* GIBBERELLIC-ACID INSENSITIVE, REPRESSOR of GAI and SCARECROW, *GTG* GPCR-type G-proteins, *Hom.* Homeostasis, *HSF* heat shock factor, *HSP-CI* small HSP class I, *JA* jasmonic acid, *LHCb* chlorophyll a–b binding protein, *LHCII* photosystem II complex, *LRR* Leucine-rich repeat receptor-like protein kinases, *LSU* large ribosomal subunit, *Met.* Metabolism, *MYB* myeloblastosis, *Mod* modification, *NAC* NAM/ATAF/CUC, *NRT1* NRT1/PTR FAMILY (NPF) proteins, *PDF2* defensin, *PHD* plant homeodomain, *PME* pectin methylesterase, *PP2C* protein phosphatase 2C, *PS* perception and signaling, *PsbS* photosystem II reaction center protein, *PYL/RCAR* receptor component PYL/RCAR, *RAV* Apetala 2/related to ABI3/VP1, *SA* salicylic acid, *SAR* systemic acquired resistance, *SDIR1* SALT- AND DROUGHT-INDUCED RING FINGER1, *SP* signaling peptides, *TPL* TOPLESS, *TPS* mono-/sesquiterpene-/diterpene synthase, *WRKY* tryptophan–arginine–lysine–tyrosine domain.

One of the most relevant results of this study is the variation of gene expression showed in needles of drought-sensitive scions (Gal 1056) grafted onto drought-sensitive (R1S) versus drought-tolerant (R18T) rootstocks, compared to the few DEGs identified in needles from drought-tolerant scions (Oria 6) grafted onto the same rootstocks (Fig. 8). DEG functional enrichment analysis revealed processes, pathways and genes that were drastically affected. In Gal 1056/R18T, upregulated DEGs were found in secondary metabolism, phytohormone action and RNA biosynthesis (Supplementary Table 2). Plants exposed to stress accumulated terpenoids and phenolic compounds like flavonoids and anthocyanins^46^, which among other roles, scavenge reactive oxygen species (ROS)^47^. Genes involved in flavonoid biosynthesis, were mostly upregulated in Gal 1056/R18T, suggesting that the drought-tolerant rootstock may be involved in flavonoid accumulation in needles from Gal 1056, while Oria 6 was not affected. Also, a significant number of genes encoding MYB TF were upregulated in Gal 1056/R18T. MYB TFs regulate different processes such as development, growth and function of organs and specific cell-types as well as metabolite biosynthesis, including flavonoids^48,49^ which could suggest that MYBs may be involved in the modulation of needle secondary metabolites content. De Miguel et al*.*^50^ identified SNPs in MYB1 TF that were associated with different concentration of phenylalanine and phenylpropanoids in *P. pinaster*. The analysis also showed DEGs associated to biosynthesis and signal transduction of phytohormones, mainly ABA and auxins, but also SA, JA, gibberellins and other signaling peptides in Gal 1056/R1S versus Gal 1056/R18T. This result suggests that rootstocks may participate in metabolism and action of these key endogenous factors that regulate plant growth and development in drought-sensitive scions (Supplementary Table 2). ABA is critical for numerous biological processes, such as bud dormancy, seed germination and plays an essential role in stress adaptation^51^. (ABA)-induced stomatal closure is modulated by different components, such as ROS, NO (nitric oxide), Ca^2+^, pH, phospholipids, K^+^, and so forth, although, to a lesser degree, ABA-independent regulation mechanisms also modulate stomatal movement^52^. Most DEGs associated with ABA perception and signaling were upregulated in Gal 1056/R1S, including genes encoding PYR/PYL receptor, known to play a major role in the regulation of stomatal opening^53^, which may be associated to pine growth as further down suggested. DEGs encoding Protein Phosphatase 2C (PP2C), a component of the PYL-PP2C-SnRK2 module, were upregulated in Gal 1056/R18T, suggesting a modification of its activity in needle cells. Finally, DEGs encoding both types of ABA transmembrane transporters, ABCG and NRT1/PTR^54^, were downregulated in Gal 1056/R18T. Auxins are involved in almost all aspects of cell division, elongation and differentiation in higher plants, and play a role in regulation and coordination of plant growth under stress^55^. DEGs related to auxin biosynthesis were upregulated, while those involved in auxin perception and signaling were downregulated in Gal 1056 grafted onto drought-tolerant rootstocks, as also were auxin transporters of ATP-binding cassette transporter superfamily^54^ (ABCB). High accumulation of flavonoid associated with the upregulation of flavonoid biosynthesis may alter auxin transport by modifying, among other mechanisms, ABCB transporter as described by Geisler & Murphy^56^.

Likewise, a significant number of genes associated to external stimuli response, photosynthesis and cell wall organization have been only found differentially expressed in Gal 1056 when grafted onto R1S versus R18T. Several downregulated genes, and therefore upregulated in Gal 1056/R1S, encoded for plant defensins, cysteine-rich proteins that mediate innate nonspecific immune response, and have been found to be involved in different abiotic stress response^57^. Also, other downregulated genes encoded components of the systemic acquired resistance (SAR), which have been recently shown to play an important role in the response to abiotic stresses, modulating reactive oxygen species, proline, and redox states^58^. Photosynthesis is another significantly enriched biological process with downregulated DEGs encoding components of Photosystem II (PSII) protein complex, including subunits of the LHCII antenna complex and PSII extrinsic proteins, such as PsbS that increases the efficiency of water use^59^. Finally, cell wall organization process included upregulated DEGs in Gal 1056/R18T, such as genes encoding caffeoyl-CoA 3-O-methyltransferase (CCoA-OMT), involved in lignin biosynthesis^60^. By contrast, genes involved in pectin metabolism such as those encoding bifunctional alpha-L-arabinofuranosidase and beta-D-xylosidase (BXL), that participate in cell wall modification^61^ and pectin methyl-esterase (PME), involved in cell adhesion^62^, were upregulated in Gal 1056/R1S. Other genes also upregulated in Gal 1056 grafted onto drought-sensitive rootstock encoded an alpha-like-class expansin, involved in cell-wall loosening and cell growth^63^ and AGP beta-1, 3-galactosyltransferase (AGP), which has a role in vegetative growth and development^64^ (Fig. 8).

Although these results point at a rootstock effect on the regulation of drought-sensitive scion transcriptome, a few DEGs were also identified in drought-tolerant scions (Oria 6) when grafted onto drought-sensitive compared to drought-tolerant rootstocks. Thus, a total of 16 DEGs were found in Oria 6 needles, mainly associated to transcriptional regulation (Supplementary Table 2). Among them, upregulated genes encoding C2H2 -ZF and bZip TFs, while downregulated genes, and therefore upregulated in Oria 6/R1S, encode for CAMTA and GRAS TFs. The most significant number of DEGs identified in Oria 6/R1S versus Oria 6/R18T encoded for CAMTA. CAMTA genes were downregulated not only in Oria 6 but also in Gal 1056, and therefore showed higher accumulation of transcripts in Oria 6/R1S and Gal 1056/R1S needles. CAMTA is a small TF family involved in calcium signaling pathway, that mediates developmental regulation and a range of responses to a variety of external and hormonal stimuli, including abiotic stresses and ABA^65^.

Considering all results discussed above, the high accumulation of transcripts associated with secondary metabolism observed in Gal 1056/R18T, the plethora of processes and pathways enriched in DEGs in Gal 1056/R1S and the practically non-existent DEGs when analyzing drought-tolerant scions, may suggest that drought-sensitive and drought-tolerant scions differ in their sensitivity to rootstock effect (Fig. 8).

Comparison between scions grafted on either drought-sensitive (Gal 1056/R1S vs. Oria 6/R1S) or drought-tolerant (Gal 1056/R18T vs. Oria 6/R18T) rootstocks provided information about processes differentially enriched in drought-tolerant (Oria 6) and drought-sensitive (Gal 1056) scions (Fig. 8). Thus, secondary metabolism was enriched in DEGs that differed between comparisons with higher number of downregulated genes involved in terpenoid and flavonoid biosynthesis, with mono-/sesqui-/diterpene synthase (ANS) and chalcone synthase (CHS) showing the most significant transcript accumulation in Gal1056/R18T. Also involved in flavonoid biosynthesis, high accumulation of flavanone 3-hydroxylase (F3H) transcripts were found in both comparisons, slightly higher in Oria 6/R1S and in Gal 1056/R18T. These results combined with the upregulation of DEGs associated to these pathways in Gal 1056/R1S versus Gal 1056/R18T and the lack of DEGs observed in Oria 6/R1S versus Oria 6/R18T indicates that Gal 1056 presents the highest activation of these pathways, that differs depending on the rootstocks they were grafted onto. These secondary metabolites are relevant compounds of plant chemical defense and act as antioxidants promoting stress tolerance^66^. Provenance-specific terpenoid patterns have been described in needles of different conifers^67^, however, this variation seems to be increased by the effect of the tolerant rootstock in drought-sensitive scions. In contrast, genes encoding gamma-glutamyl peptidase 1 (GGP1), known to be involved in glucosinolate production by cleavage of glutathione conjugate, were only upregulated in Oria 6/R18T. Glucosinolates play an important role in stomatal regulation and drought tolerance^68^. Glycine betaine biosynthesis is also enriched with upregulated DEGs only in Oria 6/R18T. Thus, our results may suggest that Oria 6, the drought-tolerant scion, showed the highest activation of these pathways when grafted onto R18T.

One of the processes that showed higher number of DEGs between Gal 1056 and Oria 6 was related to the response to external stimuli. This variation was also related to the rootstock since more upregulated genes were observed in plants grafted onto drought-sensitive rootstocks (R1S). Additionally, a relevant number of genes involved in the biosynthesis, perception and signal transduction of different phytohormones showed small variations in the expression profiles between these comparisons. Gal 1056 versus Oria 6 pines onto drought-sensitive rootstocks showed higher number of DEGs associated to auxin, mainly upregulated in Oria 6. This remarkable rootstock effect was observed in TOPLESS (TPL) that mediates the transcriptional repression of auxin pathway^69^. Regarding ABA, the gene encoding xanthoxin oxidase (ABA2), also known as xanthoxin dehydrogenase, an enzyme involved in ABA biosynthesis catalyzing the conversion of xanthoxin to abscisic aldehyde, was mainly enriched in Gal 1056 grafted onto drought-tolerant rootstocks. Additionally, DEGs encoding a salt- and drought-induced ring finger1 (SDIR1), a ring finger E3 ligase, that positively regulates stress-responsive abscisic acid signaling^70^, showed higher accumulation in Gal 1056/R18T when analyzing Gal 1056/R18T versus Oria 6/R18T. These results indicate higher accumulation of transcripts involved in ABA biosynthesis and signaling in Gal 1056/R18T which suggest activation of ABA regulation in these grafts. High transcript accumulation of PP2C, the regulatory phosphatase component of PYR/PYL complex, in Oria6/R1S may be associated with its role in plant abiotic stress tolerance by negatively regulating ABA signaling.

The comparative analysis revealed a high number of DEGs encoding a broad diversity of TFs, however, the most significant ones were MYB, AP2/ERF, C2H2-ZF, PHD, HSF, NAC, and WRKY among them key players in water stress signaling^71^. Rootstock modulation of scion transcriptome may also be supported by the expression patterns of DEGs associated to TFs. DEGs associated to C2H2-ZF, DREB and NAC were downregulated in Gal 1056/R1S versus Oria 6/R1S while upregulated in Gal 1056/R18T versus Oria 6/R18T. C2H2-ZF TFs participate in several processes during plant growth and development as well as in response to a wide spectrum of abiotic and biotic stressess^72^. NAC is a large family of TFs that is also involved in great variety of biological processes which regulate plant growth and development, as well as in abiotic stress tolerance^73^. DREB, a representative of the subfamily of AP2/ERF TFs that plays a significant role in response to drought, salinity and cold stress, also showed this trend^74^. Additionally, MYB DEGs were found in both comparisons, showing higher accumulation of upregulated transcripts in Gal 1056 and Oria 6 scions grafted onto drought-tolerant rootstocks (R18T). In contrast, DEGs encoding members of the large family of TFs AP2/ERF, key regulators of hormone and abiotic stress responses^75^, showed higher enrichment in Gal 1056 and Oria 6 scions grafted onto drought-sensitive rootstocks (R1S). DEGs encoding WRKY, a large family of TFs involved in plant development and different stress responses^76^, with members that positively or negatively regulate drought tolerance^77,78^, were upregulated in all scions but in Oria 6/R18T. Finally, two TFs that were mainly found in Gal 1056/R1S, PHD and HSF, are involved in regulating plant growth, development and response to several abiotic stresses^79^.

Other processes enriched in upregulated DEGs in Gal 1056/R1S versus Oria 6/R1S were those related to protein biosynthesis, with genes associated with ribosome synthesis, as well as with protein modification, including leucine-rich repeat receptor-like kinases (LRR-RLKs) subfamilies X and XIII. LRR-RLKs represent large number of transmembrane kinases that are involved in plant growth, development, and stress responses^80^.

In contrast, upregulated genes encoding class-C-I cytosolic small HSP were highly enriched only in Gal 1056/R18T versus Oria 6/R18T. Constitutive expression of CI sHSPs has been detected in different plant species, supporting their stress-protective role^81^. Also, genes encoding components of the light-harvesting chlorophyll-a/b proteins of photosystem II (LHCb) were enriched with upregulated DEGs. Previous QTL analysis of this full-sib family revealed the importance of maintaining the integrity of the photochemical machinery in maritime pine drought response identifying a MYB TF that was significantly associated with the efficiency of energy capture by open PSII reaction centers^26^.

Significant rootstock effect leading to higher transcript accumulation in Gal 1056/R18T was mainly associated with DEGs involved in fatty acid biosynthesis, especially upregulation of genes encoding chloroplast acetyl-CoA carboxylase (ACAC), involved in the biosynthesis of C18 unsaturated fatty acids (C18 UFAs). C18 UFAs play multiple roles such as component of membranes, reserve of carbon and energy, constituents of cutin and suberin, antioxidants, precursors of various bioactive molecules^82^, and they were recently found to be involved in signaling^83^. Considering that previous analysis of drought-sensitive and drought-tolerant siblings of genotypes used as rootstocks revealed constitutive expression of drought-related genes in tolerant pines^43^, this type of pattern may respond to the activation of C18 UFAs biosynthesis in drought-sensitive scions driven by the drought-tolerant rootstock.

## Conclusion

Grafting onto tolerant rootstocks has been amply demonstrated to improve tolerance to abiotic and biotic stresses in numerous angiosperm species. However, the molecular network behind the rootstock-scion interaction concerning drought-tolerance remains largely unknown, and almost unexplored in conifers. This study reveals processes, such as those associated to secondary metabolism, that are mainly determined by the scion genotype, as well as widespread effect of rootstocks on scion gene expression in *Pinus pinaster* (Fig. 8). The transcriptomic analysis of scion needles with contrasting drought tolerance provided information about pathways which are enriched , identifying differentially expressed genes in the grafted scion modified by drought-sensitive or drought-tolerant rootstocks. The different rootstocks significantly affected the transcript profile of Gal 1056, the drought-sensitive scion, especially the expression of genes involved in secondary metabolism, response to external stimuli, phytohormone action and RNA biosynthesis. On the contrary, gene expression pattern of Oria 6, the drought-tolerant scion, was less affected by the rootstock it was grafted onto. Drought-tolerant rootstock R18T showedaccumulation of transcripts involved in tolerance to abiotic stresses. Previous analysis of drought-sensitive and drought-tolerant siblings used as rootstocks, showed that tolerant individuals were pre‐adapted for facing drought by constitutively expressing drought‐related genes that were detected in latter stages on sensitive individuals subjected to hydric stress. Thus, our results suggest that drought-tolerant rootstocks may enhance stress tolerance in both scions by modifying the expression of genes involved in drought tolerance even under nonlimiting water conditions. However, in these grafts, processes associated with plastid activity, such as those related with photosynthesis, showed higher gene expression in drought-tolerant scions. This extensive transcriptomic analysis of rootstock effects on scion needles, has provided novel and valuable information to begin unraveling this complex interaction in conifer species. This information will also help to design graft constructs to evaluate the rootstock effect on their drought tolerance under different hydric conditions.

## Materials and methods

### Plant material and experimental design

*Pinus pinaster* grafting was performed at Centro de Mejora Genética Forestal de Valsaín (Segovia, Spain). The progenitors from a controlled cross, designed to study drought response segregation (further described in de Miguel et al.^24,26^), were used as scions. The mother tree, Gal 1056 is an elite pine from the breeding program from North-Western Spain (Pontevedra, 42° 10′ N 8° 30′ W), highly sensitive to drought and the male progenitor, Oria 6, is a tree from Sierra de Oria (Almería, 37° 31′ N 2° 21′ W), a natural population from a mountain area in South-Eastern Spain that suffers recurrent and intense droughts. Two 2-year-old siblings from this F1 full-sib family were selected for displaying contrasted response to water deficit in previous ecophysiological studies^24^ genetic characterization based on QTL analysis^26^: progeny individual 1, drought-sensitive, and progeny individual 18, drought-tolerant, which were vegetatively propagated^24^ to be used as rootstocks, R1S and R18T, respectively. Combining described scions and rootstocks, four constructions were designed: Gal 1056/R1S; Gal 1056/R18T; Oria 6/R1S and Oria 6/R18T, each one represented by three biological replicates (Fig. 1a). Top-grafting pine trees were obtained in spring and maintained in a greenhouse for 7 months.

To analyze the needle transcriptome of these grafted pines, they were transferred to a walk-in growth chamber (Fitoclima 10000EHHF, Aralab) and grown for six months, using controlled climate conditions: 14 h, 25 °C and 65% relative humidity for light period and 10 h, 20 °C and 60% relative humidity for dark period. Trees were watered to field capacity when soil volumetric water content (VWCs) dropped below 20 vol%. Different phenotypic evaluations were carried out and described in Fernández de Simón et al.^84^. Needles were harvested, frozen in liquid nitrogen, and stored at − 80 °C until RNA extraction.

### RNA extraction, RNA-Seq library preparation and sequencing

Frozen needles from each grafted pine were grinded using a MM400 Mixer Mill (Retsch GmbH and Co.) Total RNA was extracted from needle powder using Plant/Fungi Total RNA Purification Kit (Norgen Biotek Corp., Thorold. ON, Canada), following the manufacturer’s instructions. Integrity of the extracted RNA was evaluated by 1% (w/v) agarose gel analysis; concentration and quality were measured with a NanoDrop 2000 spectrophotometer (NanoDrop Technologies, Wilmington, DE). cDNA libraries were prepared using the Illumina kit TruSeq RNA library Prep. The 12 cDNA libraries were sequenced using an Illumina NextSeq 500 platform at ADM S.L. (Paterna, Valencia, Spain). Sequences were uploaded to the SRA database with Accesion ID PRJNA707426 (SRA accession numbers from SAMN8318858 to SAMN18318861).

### RNA-seq analysis

Raw reads in the Fastq format were analyzed with FastQC software (Andrews, S.: FastQC: a quality control tool for high throughput sequence data, 2010. Available online at: http://www.bioinformatics.babraham.ac.uk/projects/fastqc) and trimmed and filtered discarding low-quality reads (Q < 20) and sequences less than 30 bp using reformat.sh software (Bushnell, B., 2014), as well as ribosomal RNA using Sortmerna software^85^. Clean reads were mapped against *P. pinaster* reference transcriptome (http://www.procogen.eu) available in Plaza website^86^, that contains 206, 575 transcripts, applying a “quasi-mapping” method with kmer size > 31 pb using Salmon software^87^. Approximately 41% of the ProCoGen reference transcriptome is annotated, the remaining 122,116 transcripts may largely correspond to artifacts, non-coding sequences, putative pseudogenes due to the abundance of this type of transcripts observed in conifer genomes^88^ as well as potential conifer-specific genes and gene families^89,90^. Gene function was annotated aligning the sequences for homology searches against publicly available protein databases using BlastX implemented in the OmicsBox v1.2.4 software package^91^. Databases used were: Nr^92^ (NCBI non-redundant protein sequences); SwissProt^93^ and InterPro^94^ (classification of protein families and prediction of domains) with identity > 55% and a cutoff e-value of 10^−6^. If aligning results from different databases showed conflicted results, a priority order of alignments from SwissProt, Nr, and Interpro was followed. KEGG Ortholog^95^ (Kyoto Encyclopedia of Genes and Genomes) was used to report the molecular interaction, reaction and relation networks. OmicsBoxs v1.2.4 software was also used to obtain Gene Ontology (GO) annotations according to biological process, molecular function and cellular component, and to filter them according to Plant GO-Slim. The functions of the identified genes were evaluated by comparing with *P. pinaster* database (http://www.scbi.uma.es/sustainpine/). MapMan tool was used to visualize the functional classification of DEGs significantly affected onto diagrams of metabolic pathways^96^. Sample clustering based on the similarity of gene expression profiles of scion needles was performed using principal component analysis (PCA) using DESeq2 software^97^.

A quantitative assessment of the transcripts was used to estimate the levels of differential expression between scions grafted onto the different rootstocks, each one represented by three biological replicates (Fig. 1a). Significance levels were estimated using the Salmon and DESeq2 software, according to the procedures described by Love et al*.*^97^. Raw counts were modeling for each gene estimating size factors and gene-wise dispersions and shrinking these estimates to generate more accurate estimates of dispersion to model the counts. The statistical analysis was carried out using DESeq2’s median of ratios normalization method^98^. Genes with False Discovery Rate (FDR)-corrected *P* value (padj) < 0.05, Log2FC in count values ≤ − 1.5 or ≥ 1.5; and difference in count values > 5, were assigned as differentially expressed (DEGs).

Differential gene expression analysis pursued two main objectives: (1) the identification of genes differentially expressed in needles of each scion associated to the different rootstock they are grafted onto (Gal 1056/R1S vs. Gal 1056/R18T or Oria 6/R1S vs. Oria 6/R18T); (2) the identification of genes differentially express between Gal 1056 and Oria 6 scions grafted onto a common rootstock (Gal 1056/R1S vs. Oria 6/R1S or Gal 1056/R18T vs. Oria 6/R18T).

### Validation by quantitative real-time PCR (qRT-PCR)

Expression analysis of five DEGs was carried out by real-time qRT-PCR to validate the transcriptomic study. Gene specific primers were designed using the NCBI Primer-Blast Tool (http://www.ncbi.nlm.nih.gov/tools/primer-blast/). Selected DEGs and primer sequences are listed in Supplementary Fig. S1a. The 18S rRNA transcript was used as endogenous control for quantitative analysis. Synthesis of cDNA was performed from 1 µg of total RNA using SuperScript III First-Strand Synthesis System (Invitrogen) according to the manufacturer’s instructions. Polymerase chain reactions were performed in an Applied Biosystems 7500 Fast Real-Time PCR System (Applied Biosystems), using FastStart Universal SYBR Green Master (Rox; Roche). The reactions, containing 25 or 50 ng cDNA, 500 nM forward primer, 500 nM reverse primer and 1× SYBR Green Master, were subjected to an initial denaturation step at 95 °C for 10 min, followed by 40 cycles of 95 °C for 15 s and 60 °C for 60 s. RT-qPCR experiments were performed using three biological and three technical replicates and a melting-curve analysis was performed to verify the specificity of each primer. Relative expression was calculated by the ∆∆Ct method (Ct = threshold cycle) using 7500 Software (Life Technologies). DREB transcription factor (DREB), pre-mRNA-processing protein (LUC7), outer mitochondrion membrane TOM translocation system (TOM), C2H2-ZF transcription factor (C2H2-ZF), and macrophage migration inhibitory factor (MIF) were analyzed in needles of drought-sensitive and drought-tolerant scions (Supplementary Fig. S1).

## Supplementary information

Supplementary Information 1.

Supplementary Information 2.

Supplementary Information 3.

Supplementary Information 4.

Supplementary Information 5.

Supplementary Information 6.

Supplementary Information 7.

Supplementary Information 8.

Supplementary Information 9.

Supplementary Information 10.

## Data Availability

The Supplementary Material for this article can be found online in SRA database with Accession ID PRJNA707426 (SRA accession numbers from SAMN8318858 to SAMN18318861).
