# Supplementary material for: Rootstock effects on scion gene expression in maritime pine
Source: Sci Rep. 2021 Jun 2;11:11582. doi: 10.1038/s41598-021-90672-y (PMC8173007; doi:10.1038/s41598-021-90672-y)
Supplement: Supplementary file 1 — Supplementary Information 1. [file 41598_2021_90672_MOESM1_ESM.pdf]

Supplementary Fig. S1. Validation of sequencing data by qRT-PCR

a

Gene	Code	Transcript ID	Forward primer	Reverse primer
DREB transcription factor	DREB	unigene20075	CTCATCCCAGAAAACGTCGG	TACCAATACTTCCGGGGTGG
pre-mRNA-processing protein	LUC7	isotig26861	TTTTGACATGGGCAGTTGCG	TGAGCAGAAGTGGAACGAGAG
Outer mitochondrion membrane TOM translocation system	TOM	isotig128281	TGAGGAGCGTACTTCCGAAT	TCGATTGAGCAAGGGATTGCC
C2H2-ZF transcription factor	C2H2-ZF	unigene16552	GCCCAACTTCAGGTAAGGCA	CTTTCCTCCCCCTCTGCTTG
Macrophage migration inhibitory factor	MIF	unigene209604	TGTGACTACAACAACCTAGTTCA	ATGTCAAGTGGCACCACATTTT
Ri18S			GCGAAAGCATTTGCCAAGG	ATTCCTGGTCGGCATCGTTTA

b

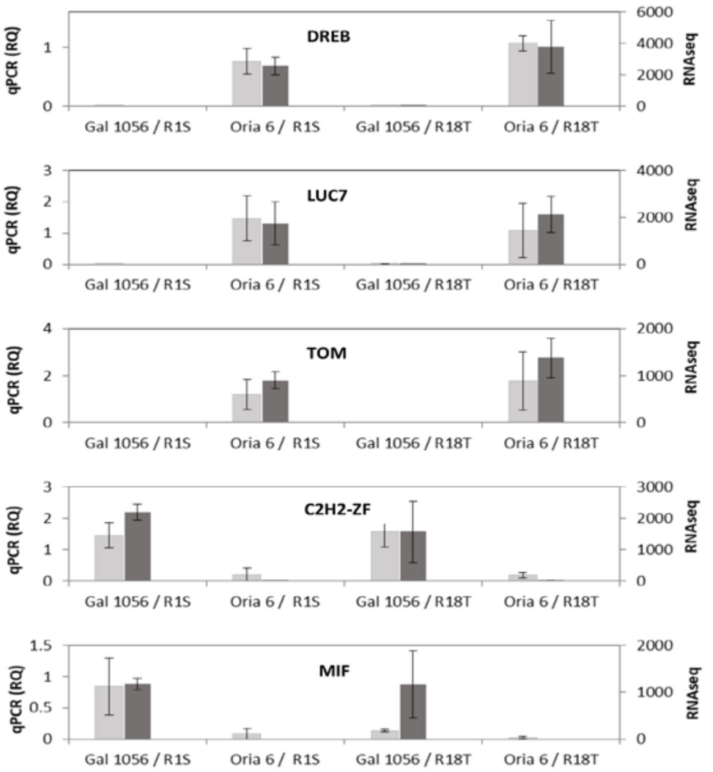

(a) Primer sequences for qRT-PCR. **(b)** Relative quantification (RQ) by qRT-PCR (grey bars) and RNA-Seq normalized expression values (black bars) of five DEGs: DREB TF (DREB), pre-mRNA-processing protein (LUC7), outer mitochondrion membrane TOM translocation system (TOM), C2H2-ZF TF (C2H2-ZF), and macrophage migration inhibitory factor (MIF)
