# Supplementary material for: Rootstock effects on scion gene expression in maritime pine
Source: Sci Rep. 2021 Jun 2;11:11582. doi: 10.1038/s41598-021-90672-y (PMC8173007; doi:10.1038/s41598-021-90672-y)
Supplement: Supplementary file 2 — Supplementary Information 2. [file 41598_2021_90672_MOESM2_ESM.pdf]

Supplementary Fig. S2. GO terms enriched in common DEGs between G/S vs O/S and G/T vs O/T

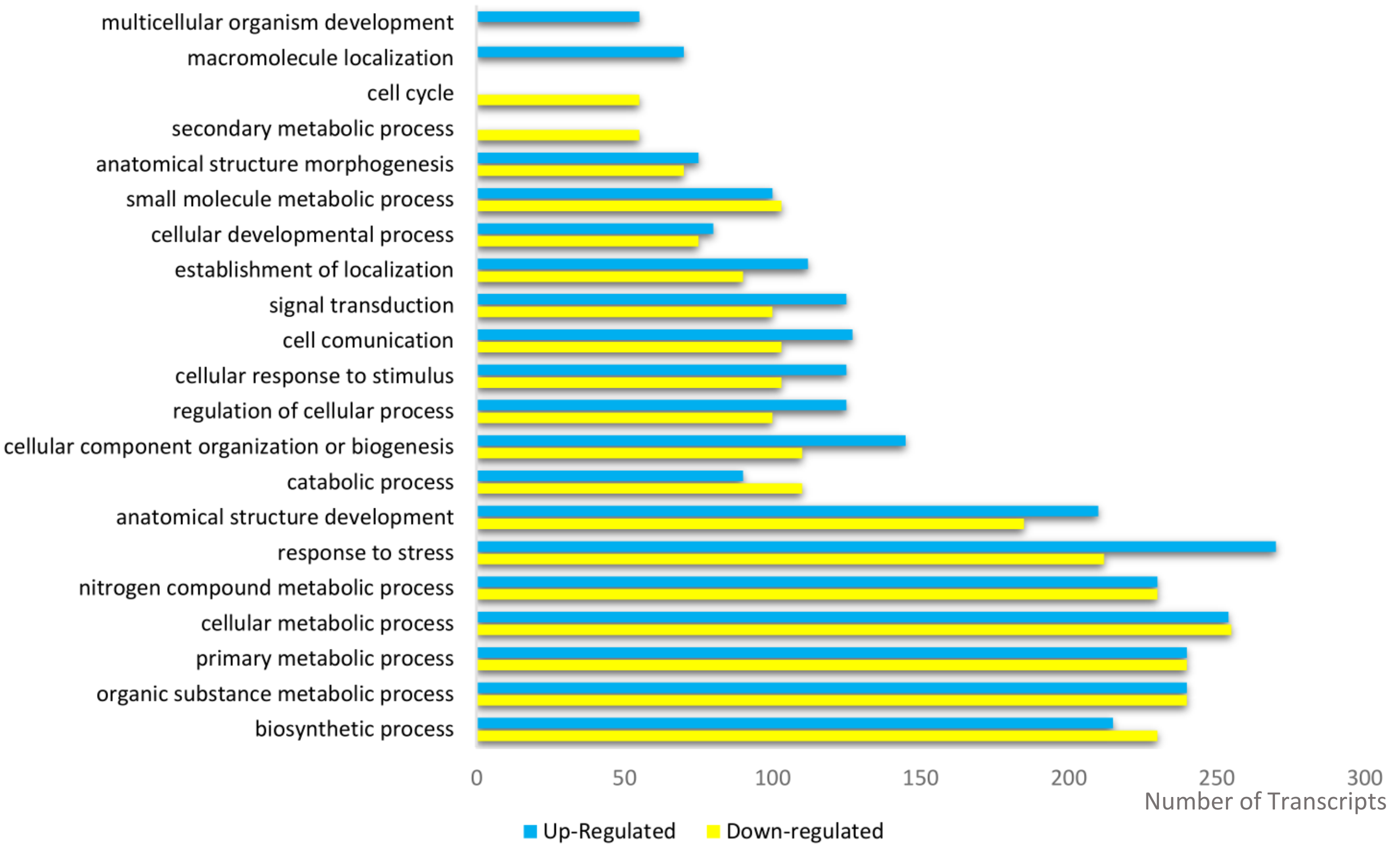

Histogram presentation of GO terms enriched in significantly upregulated (blue) and downregulated (yellow) common DEGs between G/S vs O/S and G/T vs O/T.
