# Supplementary material for: Rootstock effects on scion gene expression in maritime pine
Source: Sci Rep. 2021 Jun 2;11:11582. doi: 10.1038/s41598-021-90672-y (PMC8173007; doi:10.1038/s41598-021-90672-y)
Supplement: Supplementary file 3 — Supplementary Information 3. [file 41598_2021_90672_MOESM3_ESM.pdf]

Supplementary Table 1. Summary of *Pinus pinaster* sequencing data trimming, number of mapped reads

Genotype	Oria 6/R1S			Gal 1056/R1S			Gal 1056/R18T			Oria 6/R18T		
**Library Number**	**1**	**2**	**3**	**4**	**5**	**6**	**7**	**8**	**9**	**10**	**11**	**12**
**Raw reads (Mb)**	13.23215	12.65581	13.00199	15.99188	14.89687	16.95577	22.64033	12.65853	16.14964	16.05427	26.35359	28.38755
**Average Length**	75.51	75.52	75.54	75.52	75.52	75.53	75.52	75.56	75.55	75.53	75.52	75.53
**Average Quality**	33.89	34.45	34.40	34.27	34.52	34.45	34.45	34.33	34.31	34.36	34.42	34.45
**Reads after reformat.sh and Sortmerna processing (Mb)**	11.77858	11.62967	7.17227	12.50118	13.12024	10.90749	19.52082	4.90925	7.62297	11.48367	24.15501	22.23719
**Average Length after processing**	73	73	73	73	73	73	73	73	73	73	73	73
**Number of mapped reads (Mb)**	10.92487	10.68927	6.512493	11.37022	12.34044	10.11667	17.87468	4.19261	6.83618	10.56037	22.596	20.49219
